# Air plant genomes shed light on photosynthesis innovation

**DOI:** 10.1093/plcell/koae213

**Published:** 2024-07-24

**Authors:** Andrew C Willoughby

**Affiliations:** Assistant Features Editor, The Plant Cell, American Society of Plant Biologists; Department of Biology, Duke University, Durham, NC 27514, USA

Photosynthesis requires the uptake of CO_2_. In land plants, however, gas permeability is a double-edged sword. Opening stomata facilitates CO_2_ exchange but also allows water to evaporate. To support photosynthesis under water limitations, some plants have evolved a workaround process termed Crassulacean acid metabolism (CAM). CAM allows plants to stockpile CO_2_ at night, in the form of malate, when darkness and cooler temperatures lessen the burden of water loss through transpiration. Additionally, CAM concentrates CO_2_, which promotes the efficiency of Rubsico in producing sugars (Wickell et al. 2021). Despite the complexity required, over 30 different plant families have independently rearranged their physiology for variations of CAM (Silvera et al. 2010). The genetic features that drive the independent evolution of this metabolic process in diverse lineages are not well understood. This is especially vital as understanding the evolution of CAM may lead to strategies to increase crop resilience to drought (Borland et al. 2014).

**Figure 1.**
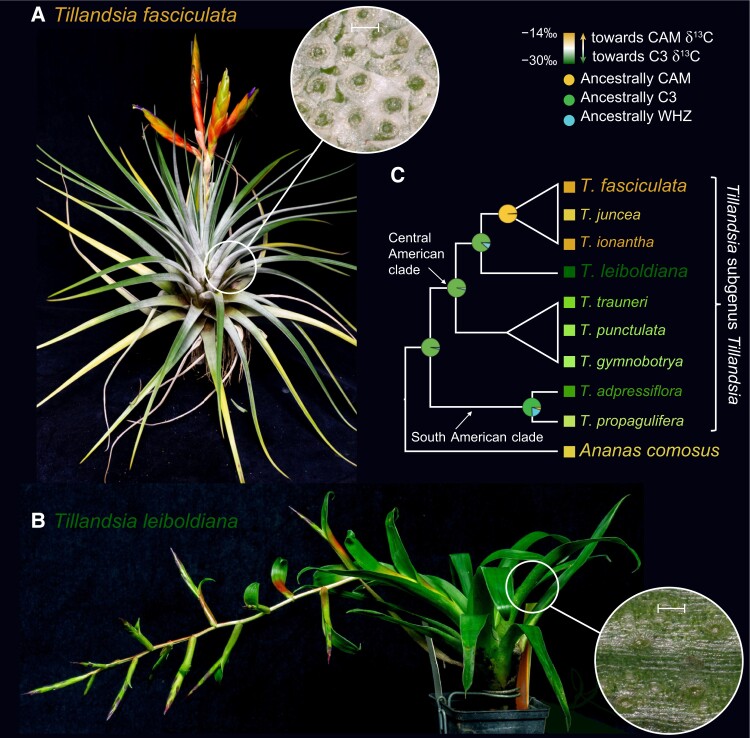
Studying CAM evolution in the genus *Tillandsia*. **A)***T. fasciculata* is a “grey” *Tillandsia* covered in a dense layer of water-absorbing trichomes (inset). **B)***T. leiboldiana* is a “green” *Tillandsia* with a water-holding tank and a reduced trichome layer (inset). **C)***T. fasciculata* and *T. leiboldiana* are closely related species that differ in their photosynthesis phenotype as measured by carbon isotope incorporation bias. C_3_ photosynthesis is biased against heavy carbon-13 isotope incorporation, and smaller negative per mille (‰) values represent carbon isotope ratios more similar to the atmosphere and therefore associated with CAM photosynthesis (De La Harpe et al. 2020). Reprinted from Groot Crego et al. 2024.

The metabolic and physiological processes of CAM support growth in arid and epiphytic conditions (Silvera et al. 2010). To study the evolution of CAM, Clara Groot Crego and colleagues (Groot Crego et al. 2024) took advantage of 2 *Tillandsia* species with divergent photosynthesis phenotypes. CAM has repeatedly evolved in *Tillandsia*, which supports the epiphytic growth found in many species in this family. Indeed, CAM is considered key to driving diversity in the Bromeliaceae and allowing the family to expand into new niches (De La Harpe et al. 2020). *T. fasciculata* is a conventional “grey” species of *Tillandsia*, named for the appearance of the dense trichomes that cover its surface and absorb water from the air (see Fig. 1A). *T. leiboldiana* is a “green” *Tillandsia* that grows in wetter environments. It collects water in a tank composed of leaves and is suggested to have conventional C_3_ photosynthesis (see Fig. 1B). These species are closely related (see Fig. 1C), and the recent evolution of CAM in *T. fasciculata* therefore makes these species an excellent model for studying CAM evolution.

Through metabolic profiling and RNAseq, the authors demonstrate that *T. leiboldiana* may be at an early stage of CAM evolution but clearly utilizes C_3_ photosynthesis. *T. leiboldiana* lacks the diurnal cycling of malate required for CAM and cycling of CAM-associated gene expression found in *T. fascicula*. The authors assembled de novo genomes of both species and analyzed them to study sequence and structural variation contributing to the evolution of CAM. *T. fascicula* exhibits selection for mutations in genes controlling trichome development and cuticular waxes, which is consistent with the phenotypic differences between “grey” and “green” *Tillandsia* species (see Fig. 1). However, the authors do not identify differential selection in the coding sequences of CAM-associated genes. Instead, they find that *T. fasciculata* has additional duplicate copies of CAM-associated genes compared with *T. leiboldiana* and that differential expression of these genes is likely driving CAM in *T. fasciculata*. Interestingly, older gene duplications shared between *T. leiboldiana* and *T. fasciculata*, that is, considered as not directly responsible for CAM in *T. fasciculata*, were nevertheless found to be overrepresented in CAM genes.

Groot Crego and colleagues describe the role of coding sequence evolution, karyotype changes, and transposable elements in shaping the genomes of *T. fasciculata* and *T. leiboldiana* and find that these changes are not driving CAM photosynthesis in *T. fasciculata*. They suggest that gene duplication in *T. fasciculata* allowed for the evolution of CAM-associated gene expression, with changes to regulatory sequences leading to subfunctionalization and neofunctionalization in the gene families required for CAM. The genome assemblies generated by the authors will be useful for future studies on CAM evolution and the Bromeliaceae (De La Harpe et al. 2020).
